# Phase II trial of AKT inhibitor MK-2206 in patients with advanced breast cancer who have tumors with *PIK3CA* or *AKT* mutations, and/or PTEN loss/*PTEN* mutation

**DOI:** 10.1186/s13058-019-1154-8

**Published:** 2019-07-05

**Authors:** Yan Xing, Nancy U. Lin, Matthew A. Maurer, Huiqin Chen, Armeen Mahvash, Aysegul Sahin, Argun Akcakanat, Yisheng Li, Vandana Abramson, Jennifer Litton, Mariana Chavez-MacGregor, Vicente Valero, Sarina A. Piha-Paul, David Hong, Kim-Anh Do, Emily Tarco, Dianna Riall, Agda Karina Eterovic, Gerburg M. Wulf, Lewis C. Cantley, Gordon B. Mills, L. Austin Doyle, Eric Winer, Gabriel N. Hortobagyi, Ana Maria Gonzalez-Angulo, Funda Meric-Bernstam

**Affiliations:** 1 Investigational Cancer Therapeutics, The University of Texas MD Anderson Cancer Center, Houston, TX 77030 USA; 2 Department of Medical Oncology, Dana Farber Cancer Institute, Boston, MA 02215 USA; 3 Columbia University, New York, NY 10027 USA; 4 Present address: Bristol-Myers Squibb, Princeton, NJ 08540 USA; 5 Biostatistics, The University of Texas MD Anderson Cancer Center, Houston, TX 77030 USA; 6 Interventional Radiology, The University of Texas MD Anderson Cancer Center, Houston, TX 77030 USA; 7 Pathology, The University of Texas MD Anderson Cancer Center, Houston, TX 77030 USA; 8 Vanderbilt University, Nashville, TN 37232 USA; 9 Breast Medical Oncology, The University of Texas MD Anderson Cancer Center, Houston, TX 77030 USA; 10 Health Services Research, The University of Texas MD Anderson Cancer Center, Houston, TX 77030 USA; 11 IND Office, The University of Texas MD Anderson Cancer Center, Houston, TX 77030 USA; 12 Systems Biology, The University of Texas MD Anderson Cancer Center, Houston, TX 77030 USA; 13 Department of Medicine, Beth Israel Deaconess Medical Center and Dana Farber Harvard Cancer Center, Boston, MA 02215 USA; 14 Cornell University, Ithaca, NY 14850 USA; 15 National Cancer Institute, Bethesda, MD 20892 USA; 16 The Sheikh Khalifa Bin Zayed Al Nahyan Institute for Personalized Cancer Therapy, The University of Texas MD Anderson Cancer Center, Houston, TX 77030 USA; 17 Breast Surgical Oncology, The University of Texas MD Anderson Cancer Center, 1400 Holcombe Boulevard, Unit 455, Houston, TX 77030 USA

**Keywords:** AKT signaling, PTEN loss, Genomics, PIK3CA mutation, Biomarkers

## Abstract

**Background:**

The PI3K/AKT pathway is activated through PIK3CA or AKT1 mutations and PTEN loss in breast cancer. We conducted a phase II trial with an allosteric AKT inhibitor MK-2206 in patients with advanced breast cancer who had tumors with PIK3CA/AKT1 mutations and/or PTEN loss/mutation.

**Methods:**

The primary endpoint was objective response rate (ORR). Secondary endpoints were 6-month progression-free survival (6 m PFS), predictive and pharmacodynamic markers, safety, and tolerability. Patients had pre-treatment and on-treatment biopsies as well as collection of peripheral blood mononuclear cells (PBMC) and platelet-rich plasma (PRP). Next-generation sequencing, immunohistochemistry, and reverse phase protein arrays (RPPA) were performed.

**Results:**

Twenty-seven patients received MK-2206. Eighteen patients were enrolled into the *PIK3CA/AKT1* mutation arm (cohort A): 13 had *PIK3CA* mutations, four had *AKT1* mutations, and one had a *PIK3CA* mutation as well as PTEN loss. ORR and 6 m PFS were both 5.6% (1/18), with one patient with HR+ breast cancer and a *PIK3CA* E542K mutation experiencing a partial response (on treatment for 36 weeks). Nine patients were enrolled on the PTEN loss/mutation arm (cohort B). ORR was 0% and 6 m PFS was 11% (1/9), observed in a patient with triple-negative breast cancer and PTEN loss. The study was stopped early due to futility. The most common adverse events were fatigue (48%) and rash (44%). On pre-treatment biopsy, *PIK3CA* and *AKT1* mutation status was concordant with archival tissue testing. However, two patients with PTEN loss based on archival testing had PTEN expression on the pre-treatment biopsy. MK-2206 treatment was associated with a significant decline in pAKT S473 and pAKT T308 and PI3K activation score in PBMC and PRPs, but not in tumor biopsies. By IHC, there was no significant decrease in median pAKT S473 or Ki-67 staining, but a drop was observed in both responders.

**Conclusions:**

MK-2206 monotherapy had limited clinical activity in advanced breast cancer patients selected for PIK3CA/AKT1 or PTEN mutations or PTEN loss. This may, in part, be due to inadequate target inhibition at tolerable doses in heavily pre-treated patients with pathway activation, as well as tumor heterogeneity and evolution in markers such as PTEN conferring challenges in patient selection.

**Trial registration:**

ClinicalTrials.gov, NCT01277757. Registered 13 January 2011.

**Electronic supplementary material:**

The online version of this article (10.1186/s13058-019-1154-8) contains supplementary material, which is available to authorized users.

## Background

PI3K/AKT/mTOR signaling plays a key role in cell growth, protein translation, autophagy, metabolism, and cell survival. Activation of PI3K/AKT/mTOR signaling contributes to the pathogenesis of many cancer types including breast cancer. Activated PI3K/AKT signaling is also associated with Cowden’s syndrome that is caused by germline phosphatase and tensin homolog (PTEN) mutations. PTEN expression is also decreased in many sporadic breast cancers. Breast cancers with increased levels of AKT phosphorylation/activation and cancers exhibiting a gene expression signature of PTEN loss show poor disease outcome [1]. Loss of PTEN activity and activation of PI3K signaling are associated with resistance to endocrine therapy [2] and trastuzumab [3, 4]. Most PTEN-low tumors and many of the phosphoinositide-3-kinase, catalytic, alpha polypeptide (*PIK3CA*) mutant tumors activate AKT for oncogenic signaling. Thus, the PI3K/AKT pathway is a promising target for breast cancer therapy.

MK-2206 is a selective allosteric inhibitor of AKT. In vitro studies of MK-2206 demonstrated that many breast cancer cell lines were highly sensitive to the agent. Preclinical studies also demonstrated that most *PIK3CA*-mutant cell lines and cell lines with PTEN loss are sensitive to MK-2206. In phase I trials, MK-2206 has been shown to inhibit AKT phosphorylation in platelets [5]. Further, serum levels of MK-2206 achieved in these trials were comparable to concentrations that achieved a growth-inhibitory effect in preclinical models. Therefore, MK-2206 was felt to hold promise as a novel AKT inhibitor.

We present here the results of a biomarker-selected phase II breast cancer trial of MK-2206. Further, we present results of correlative studies including assessment of pathway inhibition in circulating biomarkers and pre-treatment and on-treatment biopsies, as well as in-depth characterization of a patient with response to MK-2206.

## Methods

### Patient accrual

“Phase II Trial of AKT Inhibitor MK-2206 in patients with advanced breast cancer who have tumors with a *PIK3CA* mutation, or an *AKT* mutation, and/or PTEN loss/*PTEN* mutation” (NCT01277757) was a phase II trial sponsored by The Cancer Therapy Evaluation Program and conducted by the American Academy of Cancer Research Stand Up to Cancer PI3K Dream Team. This multicenter trial accrued in five centers: MD Anderson Cancer Center (lead organization), Dana-Farber Cancer Institute, Beth Israel Deaconess Medical Center, Columbia University Medical Center, and Vanderbilt-Ingram Cancer Center.

Patients with histologically or cytologically confirmed breast cancer and with metastatic/advanced disease were eligible for enrollment for screening for *PIK3CA*, *AKT*, and PTEN status after informed consent. Patients with *PIK3CA*/*AKT*/*PTEN* alterations were eligible to consent on the treatment protocol on one of two cohorts: (1) activating *PIK3CA* or *AKT1* mutations or (2) *PTEN* mutation or PTEN loss (by IHC). Although central testing was offered, patients whose tumors have already been tested in a CLIA laboratory environment and found to have a *PIK3CA* or *AKT* mutation or PTEN loss or mutation were also eligible.

From June 2011 to October 2013, 30 patients were enrolled on the trial and 27 patients received study medications. Three patients who did not initiate treatment were excluded from analysis: one withdrew consent before treatment, and two were deemed ineligible—one was found to have diffuse liver disease on ultrasound performed for pretreatment biopsy (the eligibility criteria limits liver burden to 50%), and one patient was found to have elevated liver function tests on repeat laboratory studies prior to initiating therapy.

### Study objectives

The primary objective was to determine whether MK-2206 was associated with objective tumor responses (complete response [CR], partial response [PR]) in advanced breast cancer patients with *PIK3CA* or *AKT* mutation and/or PTEN loss or mutation.

Secondary objectives included (1) determining the 6-month progression-free survival (PFS), (2) determining baseline molecular markers that may predict clinical outcome, (3) establishing pharmacodynamic markers in blood and tumor tissue that may predict outcome, and (4) determining safety and tolerability of MK-2206 in previously treated patients with advanced breast cancer.

### Treatment and evaluation plan

The patient completed any systemic therapy regimens and therapeutic radiation a minimum of 21 days prior to initiation of study therapy. Patients received MK-2206 200 mg orally once a week, until disease progression or unacceptable toxicity. Duration of each treatment cycle was 4 weeks. Dose modifications occurred per protocol according to interim toxicities. Patients underwent a pre-treatment biopsy and on-treatment biopsy on day 16 of cycle 1.

Anti-tumor efficacy was assessed using RECIST 1.1 [6]. Safety assessments were conducted at baseline, with an exam on day 15 and laboratory studies weekly during the first cycle, then day 1 of every cycle thereafter, or earlier if toxicity occurred. Toxicity was graded according to National Cancer Institute Common Terminology Criteria for Adverse Events (CTCAE), version 4.0.

### Immunohistochemistry (IHC)

Tumor biopsies (core biopsies and fine needle aspirates) were obtained pretreatment and on day 16 (1 day after third weekly treatment). IHC was performed on core biopsies. PTEN IHC was performed in the MD Anderson CLIA clinical laboratory. PTEN IHC was performed using monoclonal mouse anti-Human PTEN antibody Clone 6H2.1 from Dako at 1:100 dilution. In previous studies, we had already demonstrated that PTEN loss by IHC is associated with PI3K pathway activation [7]. PTEN staining was evaluated by assessing both intensity and percent positivity of staining. Both nuclear and cytoplasmic staining were evaluated. Staining of normal cells such as benign breast epithelium, stromal cells and/or endothelial cells was evaluated as an internal control. Any tumor nuclear or cytoplasmic staining showing similar intensity with internal control cells was considered positive staining (no PTEN loss). Complete lack of staining or faint staining (cytoplasmic or nuclear) in up to 50% of tumor cells was considered as PTEN loss. If there was no staining in internal control cells, the staining was considered to be inconclusive.

Pre-treatment and on-treatment core biopsies were also assessed for pAKT Ser473 (1:50), pS6 Ser235/236 (1:50), and pS6 Ser240/244 (1:200; all from Cell Signaling, Danvers, MA) as previously described [7]. The Refine Polymer Detection kit was used for immunostaining, with 3,3-diaminobenzidine serving as chromagen. Slides were counterstained with Mayer’s hematoxylin. Antibodies were evaluated with known positive and negative tissue controls.

### Reverse phase protein arrays

Samples were evaluated by reverse phase protein arrays (RPPA) to assess PI3K activation status as previously described [7–9]. Fine-needle aspiration biopsy samples were obtained at pre- and post-treatment from patients and were frozen immediately. Among 24 patients who had biopsies, one patient was excluded because there was no tumor in samples for H&E analysis of core biopsies. Fifteen patients had paired pre- and post-treatment samples available, while eight patients had pre-treatment samples only. Peripheral blood mononuclear cells (PBMCs) and platelet enriched plasma (PRP) were evaluated by RPPA at the following time points: at screening, C1-D1 (pretreatment), C1-D1 (post-treatment), and C1-D2.

The RPPA raw data were normalized by loading control by the RPPA core and log2 transformed. A linear mixed effects (LME) model was used to assess the differences in protein expression between time points on a protein-by-protein basis. The LME model includes the fixed effect of time point (two pre-treatment: early, C1D1: pre-treatment; two post-treatment: C1D1: post-treatment, C1D2) and random effect of the patient. To account for multiple testing, we estimated the false discovery rates (FDR) of the overall test of the model using the Benjamini-Hochberg method [10]. The modified *Z*-scores of 9 important PI3K pathway biomarkers were calculated and used to compute the composite PI3K pathway activity score. The modified *Z*-score proposed by Iglewicz and Hoaglin [11] was calculated based on the median of expression and absolute deviation about the median. We defined the patient’s composite PI3K pathway activity score as the sum of the modified *Z*-scores of phospho-protein of pAKT, 4E-BP1, S6K, and S6 (i.e., PI3K score = pS6 S240/244 + pS6 S235/236 + pS6K T389 + p4E-BP1 S65 + p4E-BP1 T37/46 + p-mTOR S2448 + pPRAS40 T246 + pAKT S473 + pAKT T308). The LME model described above was used to compare the PI3K activity scores between pre- and post-treatment.

### DNA and RNA analysis

CLIA DNA analysis was performed initially using the Sanger sequencing, and then transitioned to mass spectroscopy-based multiplex assay to assess the mutational status of hotspot regions in 11 genes (Sequenom) or with next-generation sequencing using the Ion Ampliseq 46 Gene Cancer Panel (Life Technologies) to assess hotspot mutations in *PIK3CA* and *AKT1* genes as previously described [12]. Through the course of the trial, genomic testing became more comprehensive and frequently performed at individual institutions. Testing performed on alternate CLIA platforms was allowed. Genomic testing was performed on available archival tissue; testing on either primary tumors or metastatic biopsies was allowed, but testing on most recent samples was encouraged.

Targeted exome sequencing was performed on the DNA extracted from pre-treatment biopsies using a 202 gene platform (T200), and previously described methodology [13].

### Statistical considerations for clinical outcomes

We used a Bayesian adaptive phase II design for single-agent MK-2206 in advanced breast cancer. The primary endpoint was tumor response (complete or partial response). There were two cohorts of patients, *PIK3CA/AKT1* mutant vs. PTEN loss; we expected antitumor activity in both cohorts.

### Preclinical modeling

1 × 10^7^ ZR75-1 breast cancer cells were inoculated in the mammary fat pads of female nu/nu mice (Department of Experimental Oncology, MD Anderson). Mice were subcutaneously implanted with 17β-estradiol pellets (Innovative Research of America). Mice bearing ZR75-1 xenografts were randomized into 3 groups (vehicle, MK-2206 240 mg/kg, or 480 mg/kg, *n* = 5–6). Tumor measurements were followed to assess antitumor efficacy, and RPPA was utilized to assess the effect on cell signaling as described above. All animal experiments were approved by the MD Anderson Cancer Center Animal Care and Use Committee.

## Results

### Patient characteristics

Table 1 summarizes patient characteristics. The median age was 51 years (range 30–73 years). Fifteen patients had ER or PR-positive disease (HR-positive), three had HER2-positive disease, and nine had triple-negative breast cancer (TNBC). A median number of previous lines of therapy was six (range 2–9). Four patients had previously received everolimus.

**Table 1:** Patient and tumor characteristics by study cohort

Characteristic	Total patients (*n* = 27)	PIK3CA/AKT cohort (*n* = 18)	PTEN cohort (*n* = 9)
Age			
Mean (range)	52 (30–73)	54 (33–73)	48 (30–68)
Race			
Caucasian	24 (89%)	16 (89%)	8 (89%)
African-American	3 (11%)	2 (11%)	1 (11%)
ECOG performance status			
0	19 (70%)	13 (72%)	6 (67%)
1	8 (30%)	5 (28%)	3 (33%)
Subtype			
HR+	15 (56%)	12 (67%)	3 (33%)
TNBC	9 (33%)	4 (22%)	5 (56%)
HER+	3 (11%)	2 (11%)	1 (11%)
Molecular subtype			
*PIK3CA* mutant	13 (48%)	13 (72%)	–
*AKT1* mutant	4 (15%)	4 (22%)	–
*PIK3CA* mutant and PTEN loss	1 (4%)	1 (6%)	–
PTEN loss	9 (33%)	–	9 (100%)
Number of prior therapies			
2	2 (7%)	1 (5%)	1 (11%)
3	3 (11%)	1 (5%)	2 (22%)
≥ 4	22 (82%)	16 (90%)	6 (67%)

Twenty-seven patients received MK-2206 treatment: 18 patients were treated on the *PIK3CA/AKT1* mutation arm (cohort A)—13 had *PIK3CA* mutations, 4 had *AKT1* mutations, and one had a *PIK3CA* mutation as well as PTEN loss. Nine patients were enrolled on the *PTEN* mutation/loss arm (cohort B): 4 with *PTEN* mutations and 5 with PTEN loss by IHC.

### Antitumor activity

Twenty-seven patients who received treatment were considered in the efficacy and toxicity assessments. The study used an adaptive two-stage design stratified according to the cohort. In stage I of the study, we observed a confirmed partial response in a patient with a breast cancer bearing a *PIK3CA* mutation. By our adaptive design, one objective response fulfilled stage I criteria for response, thus both *PIK3CA/AKT* and PTEN loss/mutation cohorts proceeded to accrue. If there were no further responses seen, based on the initial statistical plan, we would be expected to treat 20 evaluable patients on the *PIK3CA/AKT* arm and 12 patients on the PTEN loss arm before clinical futility would be declared and trial terminated. However, after 27 patients were enrolled in the study, no additional objective responses were seen, and the median PFS was 8 weeks. Therefore, the clinical team, after consulting with the trial statistician, made a decision to stop accrual due to a lack of clinical benefit.

**Fig. 1:**
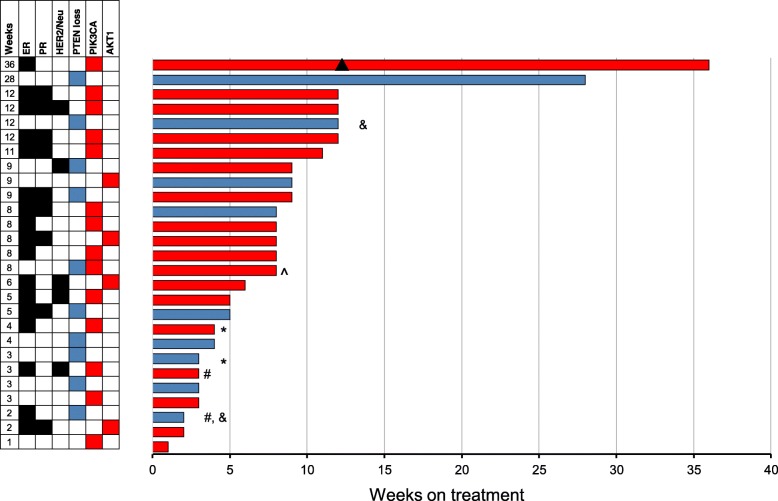
Individual swimmer plots for each patient in the overall study population. Depicting progression-free survival (PFS) for PTEN cohort (blue), and PFS for PIK3CA/AKT cohort (red). Patients who discontinued MK-2206 due to toxicity are depicted with a (#), while patients who discontinued MK-2206 due to patient choice are depicted with a (*). Two patients who were enrolled on the PTEN loss cohort had PTEN expressions on the pre-treatment biopsy sample are depicted with a (&). A patient who had both PTEN loss and *PIK3CA* mutation is depicted with a (^). One patient who had partial response starting at 12 weeks (▲)

Figure 1 shows the swimmer plots for 27 patients. Eighteen patients were enrolled in the *PIK3CA/AKT* cohort. Of 14 patients with *PIK3CA* mutations, one patient had a partial response (described below). Two patients came off the study due to toxicity and did not have an objective response assessment. One patient with both a *PIK3CA* mutation and PTEN loss was assigned to the *PIK3CA/AKT* arm and did not respond to treatment. Four patients with *AKT* E17K mutations were also enrolled with no objective responses. The median PFS was 8 weeks for this cohort.

The patient who had a confirmed partial response in the *PIK3CA/AKT* cohort had metastatic ER+, PR-, and HER2- breast cancer with a *PIK3CA* E542K mutation. Interestingly this patient also had a *KRAS* G12S mutation. She remained on study for 9 cycles (36 weeks). The patient was subsequently treated with exemestane and everolimus but progressed within 3 months.

Nine patients were enrolled in the PTEN loss/mutation cohort: Two of these patients could not be assessed for objective response: one elected to come off treatment due to rash within the first cycle, while the second patient came off the study after 3 doses due to patient choice (elected treatment closer to home). Of the seven patients who were evaluable for response, none had an objective response and only one had SD > 6 months (28 weeks).

### Safety and tolerability

Patients who received at least one dose of MK-2206 were considered eligible for toxicity assessment. The most common adverse events were fatigue (48%), rash (44%), vomiting (30%), and nausea (28%). (Table 2). As previously reported, rash was a common finding and seven patients had grade 3 rash: one patient elected to discontinue treatment, and two had dose reductions.

**Table 2:** Treatment-related adverse events reported in ≥ 10% of patients (*n* = 27)

Drug-related AE	All grades *N* (%)	Grade 3 *N* (%)
Fatigue	13 (48%)	4 (15%)
Rash	12 (44%)	7 (26%)
Vomiting	8 (30%)	0
Nausea	7 (26%)	0
Diarrhea	7 (16%)	0
Pain	5 (19%)	1 (4%)
AST increased	4 (15%)	2 (7%)
Hyperglycemia	4 (15%)	1 (4%)
Pruritus	4 (15%)	1 (4%)
Anorexia	4 (15%)	0
Mucositis	4 (15%)	0
Insomnia	3 (11%)	0
Anemia	3 (11%)	0
Sore throat	3 (11%)	0
Dysgeusia	3 (11%)	0
Constipation	3 (11%)	0

One subject was admitted for lethargy and fever 41 days after initiation of protocol therapy. The last date of MK-2206 therapy was administered 5 days prior to hospital admission. Laboratory evaluation was notable for hematocrit 10 (baseline 30), platelet count 12,000 (baseline 110,000), mild elevation of creatinine, and hyperbilirubinemia. Peripheral smear was consistent with microangiopathic hemolytic anemia (MAHA), as well as the presence of nucleated red blood cells and early myeloid forms, suggestive of marrow infiltration by her widely disseminated breast cancer. Coagulation studies were consistent with chronic DIC. A diagnosis of cancer-associated MAHA was made, and the event was deemed probably attributable to disease progression and possibly related to treatment with MK-2206. She expired on day 6 of hospital admission, 47 days after the start of protocol therapy.

### Circulating markers

**Fig. 2:**
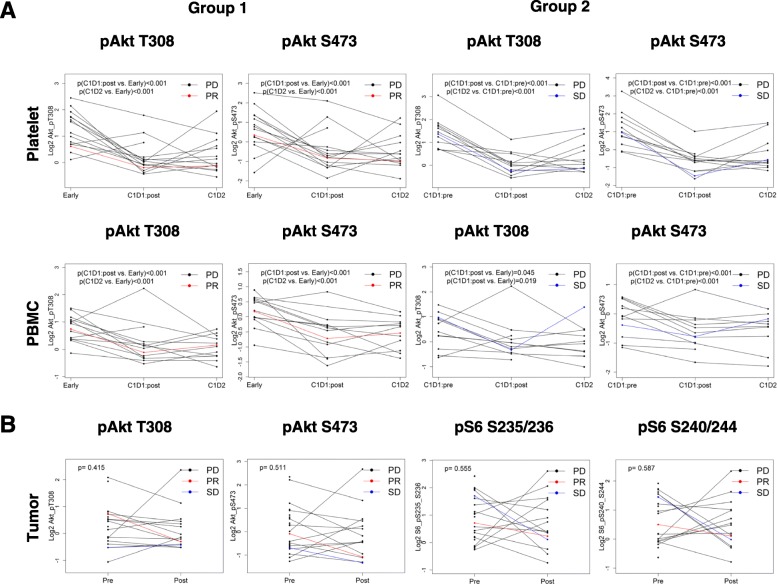
The effect of MK-2206 on signaling in PBMC and PRP samples. Differences between baseline and post-treatment expression of pAKT-T308, pAKT-S473, pS6 S235/236 and pS6 S240/244 with two-sided *t* test. *P* < .05 was considered statistically significant. **a** Upper panel—platelets: group 1: early and C1D1: post-treatment, early and C1D2 for 15 patients who have early samples available. Group 2: C1D1:pre-treatment and C1D1: post-treatment, C1D1:pre-treatment and C1D2 for 13 patients who have C1D1:pre-treatment available. Lower panel—PBMC: group 1: early and C1D1:post-treatment, Early and C1D2 for 15 patients who have early samples available Group 2: C1D1:pre-treatment and C1D1:post-treatment, C1D1:pre-treatment and C1D2 for 13 patients who have C1D1:pre-treatment available. **b** Tumor

In previous work, we studied breast cancer xenografts with increasing doses of MK-2206 and observed a dose-dependent decrease in tumor growth (including tumor regression) and decreases in PI3K signaling including pAKT S473 and pAKT T308 (see Additional file 1: Figure S1) [14]. In order to determine the effect of MK-2206 on signaling, we collected pre-treatment and post-treatment PBMC and PRP samples from patients. Post-treatment C1D1 and C1D2 samples were available in 26 patients. In 13 patients we also had C1D1 pretreatment samples, while in the other 15 we only had samples that had been collected at baseline (during screening). Two patients had both early (screening) and C1D1 pre-treatment samples available. For the 15 patients who had baseline PRP samples available, expression of pAKT S473 and pAKT T308 significantly decreased from baseline to C1D1 post-treatment (*p* = 0.001 and *p* < 0.001 respectively) and from baseline to C1D2 (both *p* < 0.001; Fig. 2a upper panel group 1). Using the LME model which includes fixed effect of time point and random effect of the patient, the difference in PI3K pathway activity scores between baseline and C1D1 post-treatment and baseline and C1D2 demonstrated a statistically significant decrease (Fig. 2a upper panel). Similarly, for 13 patients who had C1D1 pre-treatment PRP samples available, the expression of pAKT S473 and pAKT T308 significantly decreased from C1D1-pre-treatment to C1D1-post-treatment (both *p* < 0.001) and C1D2 (both *p* < 0.001; Fig. 2a upper panel group 2). The PI3K pathway activation score also significantly decreased (*p* < 0.001).

Similarly, within patients who had early PBMC samples available, expression of pAKT S473, pAKT T308, and PI3K pathway activity score significantly decreased from baseline to C1D1-post-treatment and C1D2 (Fig. 2a lower panel group 1). Among patients who had C1D1-pre-treatment samples available, the expression of pAKT S473 significantly decreased from C1D1-pretreatment to C1D1-post-treatment and to C1D2 (Fig. 2a lower panel group 2).

### Pharmacodynamics in tumor tissue

Fifteen patients had matched pre-treatment and on-treatment biopsy samples for RPPA. The difference between samples was not significant for pAKT T308 (*p* = 0.415), and pAKT S473 (*p* = 0.511). Of the 220 markers assessed by RPPA only eEF2 was significantly down-regulated after treatment (fold change − 1.18; *p* = 0.028).

**Fig. 3:**
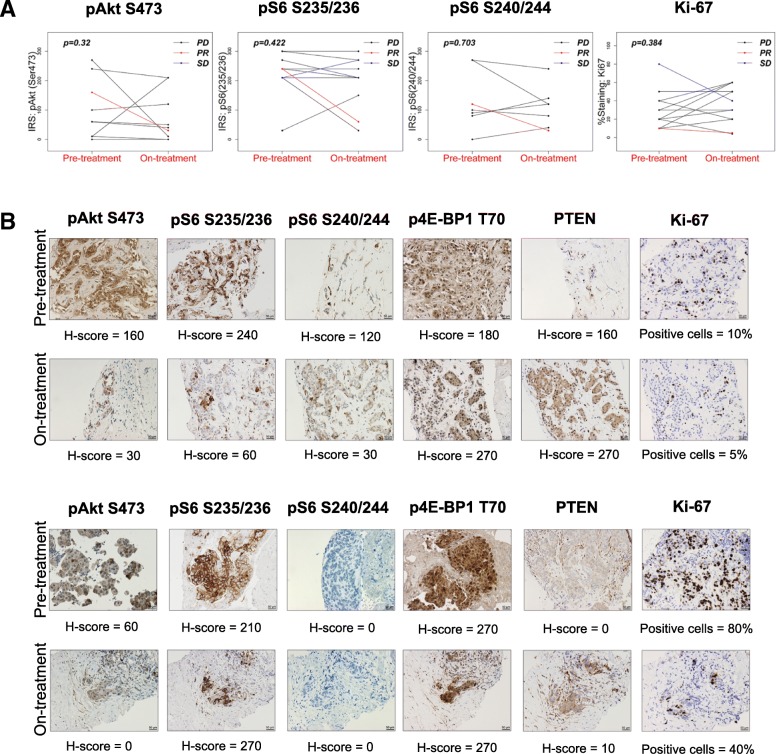
Expression of PI3K/AKT/mTOR signaling pathway markers in paired baseline and on-treatment samples. **a** IRS score for pAKT S473, pS6 S235/236, pS6 S240/244, and Ki-67 in paired baseline and on-treatment samples. **b** Expression of PI3K/AKT/mTOR signaling pathway markers in the two patients who demonstrated clinical benefit. Upper panel, patient with partial response in PIK3CA cohort; bottom panel, patient with stable disease in PTEN cohort

PI3K pathway activation was also assessed by IHC for pAKT S473, pS6 S235/236, pS6 S240/244, and p4EBP1 T70 (Fig. 3). Overall in the study, no difference in IRS score was observed between paired baseline and on-treatment samples based on Wilcoxon signed-rank tests (Fig. 3a). Notably, IHC did reveal pathway inhibition in the two patients who evidenced clinical benefit (Fig. 3b).

### Sequencing and PTEN IHC

Pre-treatment biopsies were available for hybrid-capture next-generation sequencing (NGS). Of the patients who had pre-treatment biopsies, all patients known to have *PIK3CA*, *AKT1*, or *PTEN* mutations based on CLIA testing on archival tissue were confirmed to have the same alterations on NGS of the pre-treatment biopsy. Of the patients who had evaluable samples with confirmation of their *PIK3CA* mutations, the following *PIK3CA* single nucleotide variations were found: H1047R (*n* = 8, with one patient with an additional E365K mutation), E542K (*n* = 8), E545Q (*n* = 1), and W545Q (*n* = 1). Additional file 2: Table S1 shows the targeted sequencing data from the two best responders in the study cohort.

Pre-treatment and on-treatment biopsies were assessed for PTEN expression by IHC. Of the 22 patients with evaluable pre-treatment biopsies, 19 (86%) had PTEN results concordant with pre-treatment assessment on archival samples. In contrast, three patients had discordant results. Two of the five patients who were enrolled on the PTEN loss cohort based on archival testing had PTEN expression on the pre-treatment biopsy sample, and one patient who was enrolled on *PIK3CA* cohort had PTEN expression on archival analysis but had PTEN loss on pre-treatment analysis. Ten patients had evaluable pre-treatment and on-treatment biopsies. Three patients had discordant results between pre- and on-treatment biopsies; two patients with PTEN expression at baseline lost expression with treatment, while one patient with PTEN loss had upregulation of PTEN expression. Thus, in the small number of patients enrolled on this trial, there was significant discordance in IHC results in different biopsies.

## Discussion

There is wide recognition that PI3K/AKT/mTOR signaling plays a critical role in many cancer types including breast cancer. We therefore conducted this Phase II trial with the AKT Inhibitor MK-2206 in patients with advanced breast cancer who had tumors with a *PIK3CA* mutation or an *AKT* mutation and/or PTEN loss/*PTEN* mutation. We observed that MK-2206 monotherapy had the expected toxicity profile, with a substantial incidence of grade 3 rash, but had limited clinical and pharmacodynamic activity as monotherapy.

In our preclinical work with MK-2206, we had demonstrated that *PIK3CA*-mutant cell lines and cell lines with PTEN loss were more sensitive to MK-2206 [14]. In this study, however, in patients selected with these genomic alterations, we saw limited clinical activity. Our patients were heavily pretreated and this may have affected the efficacy signal. However, it is notable that although we saw a pharmacodynamic effect in PRP, we did not see significant pathway inhibition in our tumor samples. In our preclinical work, we had shown that MK-2206 led to a dose-dependent decrease in AKT signaling in vitro and a dose-dependent decrease in tumor growth in vivo, with regression observed at higher doses that were associated with decreases in AKT signaling. These results are in line with a recent report from Kalinsky et al. where they assessed MK-2206 pharmacodynamics effect in a presurgical window-of-opportunity trial in breast cancer [15]. Two doses of MK-2206 were administered, 9 and 2 days before surgery. In spite of dose reductions, the trial was discontinued after 12 patients were enrolled due to toxicity being greater than expected (adverse events of rash and mucositis). While there was a trend of reduction in pAkt with treatment, this did not reach statistical significance and did not represent a significant change from controls. There were no significant changes in Ki-67 or pS6. Taken together, the data suggests that unlike the xenograft experiments, in patients, toxicities and other patient and tumor-related factors limit the dose delivery, leading to insufficient target inhibition in the tumor, and thus limiting antitumor activity. Notably, although rash was a common adverse event, the trial did not decrease the starting dose due to concerns that decreasing dose might decrease efficacy. Both patients that had clinical benefit from MK-2206 were able to tolerate 200 mg weekly dosing of MK-2206 with limited rash and had evidence of pathway inhibition in the tumor.

Recently, several clinical trials with MK-2206 have been reported. There has been limited efficacy associated with MK-2206 monotherapy in renal cell carcinoma [16] and modest activity in lymphoma [17]. There has been some signal of activity with combination therapies such as MK-2206 with trastuzumab [18] and MK-2206 with ridaforolimus [19]. In a randomized phase II trial of a combination of MK-2206 with selumetinib, the combination was associated with higher adverse event rate and lower efficacy compared to modified FOLFOX in metastatic pancreatic cancer [20].

The combination of MK-2206 (150 mg weekly) and anastrozole was assessed by Ma et al. in a neoadjuvant trial in 16 patients with *PIK3CA* mutant HR+ breast cancer [21]. Three patients were taken off-study due to on-treatment Ki67 > 10% (*n* = 2) and toxicity (*n* = 1). Thirteen patients completed neoadjuvant therapy followed by surgery; none had a pathologic complete response. Addition of MK-2206 decreased pAKT S473 but did not decrease pPRAS40 T246 and did not further decrease Ki-67. The two patients that went off study due to inadequate Ki67 suppression also did not have adequate pAKT S473 suppression. Thus, overall MK-2206 did not appear to adequately suppress downstream signaling or proliferation, and thus unlikely to add to the efficacy of anastrozole alone.

MK-2206 has also been explored in combination with paclitaxel. In a Phase I trial, twenty-two patients were treated with combination therapy, 9 in dose escalation (multiple tumor types) and 13 in dose expansion in breast cancer [8]. There were five objective responses, and nine patients had stable disease. None of the patients with responses had *PIK3CA, AKT* or *PTEN* mutations. MK-2206 (135 mg po daily) was also tested in combination with paclitaxel in the I-SPY trial [22]. MK-2206 improved predicted pCR rates compared to standard chemotherapy in several breast cancer signatures, defined mostly by HR− and HER2+ patients, with predicted pCR rates with and without MK-2206 being 64.1% and 35.7% in HR−/HER2+ and 46.7% and 26.1% in HR−/HER2− patients respectively.

There are several ongoing clinical trials with other novel AKT inhibitors including allosteric inhibitors ARQ092, BAY1125976, TAS-117, catalytic inhibitors AZD5363, ipatasertib, GSK2141795, and the dual AKT/S6K inhibitor MSC2363318A. Recently, Hyman et al. reported AZD5363 monotherapy data in patients with AKT alterations [23]. In this study of 52 *AKT1* E17K–mutant patients treated with AZD5363, confirmed partial responses were observed among patients with HR+ breast and endometrial cancers (*n* = 4 and *n* = 2, respectively), as well as cervical cancer, triple-negative breast cancer, and lung adenocarcinoma (*n* = 1 each), with four additional unconfirmed partial responses. Additionally, the PAKT trial reported that the addition of AZD5363 to first-line paclitaxel therapy for TNBC resulted in significantly longer PFS (5.9 months vs. 4.2 months) and overall survival (19.1 months vs. 12.6 months) [24]. The LOTUS trial investigated the addition of ipatasertib to paclitaxel as first-line therapy for triple-negative breast cancer [25]. Median progression-free survival in the intention-to-treat population was 6.2 months (95% CI 3.8–9.0) with ipatasertib versus 4.9 months (3.6–5.4) with placebo (stratified hazard ratio [HR] 0.60, 95% CI 0.37–0.98; *p* = 0.037). Pre-specified analyses in the subgroup of 42 patients with *PIK3CA/AKT1/PTEN*-altered tumors, after PFS events in 12 (46%) of 26 patients in the ipatasertib group and 13 (81%) of 16 patients in the placebo group, showed median PFS of 9.0 months with ipatasertib versus 4.9 months with placebo (non-stratified HR 0.44, 95% CI 0.20–0.99, log-rank *p* = 0.041). In patients with *PIK3CA/AKT1/PTEN-*non-altered tumors, the PFS was not significantly different. Therefore, there is emerging data that AKT is indeed an effective target for cancer therapy in breast cancer. Further studies are needed to demonstrate whether newer generation agents such as ipatasertib or AZD5363 achieve greater target inhibition, either due to their mechanism of action (catalytic vs allosteric inhibitors) or due to better tolerability.

Our study had several limitations. We studied weekly dosing of MK-2206 as we expected that weekly dosing would be more tolerable than daily dosing; thus, we cannot exclude that more frequent dosing and thus more continuous inhibition would not have been more effective. We also did not obtain pharmacokinetic data; however, we used a dose where pharmacokinetics have previously been studied [26]. We included tumors of different breast cancer subtypes and heavily pre-treated patients, and this may have dampened efficacy signals. We did not collect information on white blood cell count or other immune variables. Additionally, we initiated the trial at a time when genomic testing was less frequently offered in clinical practice, which created problems with timely accrual of patients to the study. Of note, as the frequency and availability of genomic testing increased, during the course if the trial, patients were enrolled based upon local testing on many different platforms, even within the same institutions. Finally, we enrolled patients based on archival testing results but the discordances between the PTEN status on archival tissue and that from pre-treatment biopsies highlights the drawback of enrollment based on archival testing, an approach used in most biomarker-selected trials. Although we were successful in getting pre-treatment and on-treatment biopsies, the sample size and the limited number of patients with clinical benefit limits our ability to make significant correlations with the outcome.

## Conclusions

In conclusion, MK-2206 monotherapy had limited clinical activity in advanced breast cancer patients selected for *PIK3CA/AKT1* or *PTEN* mutations or PTEN loss. This may, in part, be due to inadequate target inhibition at tolerable doses in heavily pre-treated patients with pathway activation, as well as tumor heterogeneity and evolution in markers such as PTEN conferring challenges in patient selection. The lack of statistically significant pathway inhibition in the tumors was an interesting finding that needs further study, and raises awareness that circulating biomarkers may not adequately reflect target inhibition in tumor tissue. The fact that one of the patients with a PR had an activating *KRAS* mutation, also confirmed on pre-treatment biopsy, is unexpected, and suggests that the simplistic view that MAPK co-mutations would confer resistance to AKT inhibitors may not be sufficient for precision oncology, and better modeling of driver mutations, pathway activation, and adaptive responses is needed.

## Additional files

Additional file 1: **Figure S1.** MK-2206 inhibits tumor growth in ZR75-1 xenografts. A. Scatter plots show the change in tumor volume (TV) calculated for each tumor using the formula (Vf-V0)/V0, where V0 is initial volume (at the beginning of treatment) and Vf is final volume (at the end of treatment). Lines are at mean with SD. Treatment groups (MK-2206 dosed at 240 or 480 mg/kg) were compared with the vehicle (30% Captisol (CYDEX Pharmaceuticals)). B. Heatmap of the 13 significant dose-dependent proteins at the FDR of 0.1 in ZR-75-1 xenografts. (PPTX 130 kb)

Additional file 2: **Table S1.** Sequencing data from a 202 gene platform (T200) for two best responders in the study cohort. (DOCX 18 kb)

## Abbreviations

6 m PFS: 6-month progression-free survival
CR: Complete response
CTCAE: Common Terminology Criteria for Adverse Events
FDR: False discovery rates
HR+: Hormone receptor positive
IHC: Immunohistochemistry
LME: Linear mixed effects
ORR: Objective response rate
PBMC: Peripheral blood mononuclear cells
PFS: Progression-free survival
PIK3CA: Phosphoinositide-3-kinase, catalytic, alpha polypeptide
PR: Partial response
PRP: Platelet-rich plasma
PTEN: Phosphatase and tensin homolog
RPPA: Reverse phase protein arrays
TNBC: Triple-negative breast cancer

## References

[1] Saal LH, Johansson P, Holm K, Gruvberger-Saal SK, She QB, Maurer M, Koujak S, Ferrando AA, Malmstrom P, Memeo L (2007). Poor prognosis in carcinoma is associated with a gene expression signature of aberrant PTEN tumor suppressor pathway activity. Proc Natl Acad Sci U S A.

[2] Miller TW, Perez-Torres M, Narasanna A, Guix M, Stal O, Perez-Tenorio G, Gonzalez-Angulo AM, Hennessy BT, Mills GB, Kennedy JP (2009). Loss of phosphatase and tensin homologue deleted on chromosome 10 engages ErbB3 and insulin-like growth factor-I receptor signaling to promote antiestrogen resistance in breast cancer. Cancer Res.

[3] Nagata Y, Lan KH, Zhou X, Tan M, Esteva FJ, Sahin AA, Klos KS, Li P, Monia BP, Nguyen NT (2004). PTEN activation contributes to tumor inhibition by trastuzumab, and loss of PTEN predicts trastuzumab resistance in patients. Cancer Cell.

[4] Berns K, Horlings HM, Hennessy BT, Madiredjo M, Hijmans EM, Beelen K, Linn SC, Gonzalez-Angulo AM, Stemke-Hale K, Hauptmann M (2007). A functional genetic approach identifies the PI3K pathway as a major determinant of trastuzumab resistance in breast cancer. Cancer Cell.

[5] Yap TA, Yan L, Patnaik A, Fearen I, Olmos D, Papadopoulos K, Baird RD, Delgado L, Taylor A, Lupinacci L (2011). First-in-man clinical trial of the oral pan-AKT inhibitor MK-2206 in patients with advanced solid tumors. J Clin Oncol.

[6] Eisenhauer EA, Therasse P, Bogaerts J, Schwartz LH, Sargent D, Ford R, Dancey J, Arbuck S, Gwyther S, Mooney M (2009). New response evaluation criteria in solid tumours: revised RECIST guideline (version 1.1). Eur J Cancer.

[7] Meric-Bernstam F, Akcakanat A, Chen H, Sahin A, Tarco E, Carkaci S, Adrada BE, Singh G, Do KA, Garces ZM (2014). Influence of biospecimen variables on proteomic biomarkers in breast cancer. Clin Cancer Res.

[8] Gonzalez-Angulo AM, Krop I, Akcakanat A, Chen H, Liu S, Li Y, Culotta KS, Tarco E, Piha-Paul S, Moulder-Thompson S, et al. SU2C phase Ib study of paclitaxel and MK-2206 in advanced solid tumors and metastatic breast cancer. J Natl Cancer Inst. 2015;107(3):dju493. https://www.ncbi.nlm.nih.gov/pubmed/25688104. 10.1093/jnci/dju493 PMC4342675 25688104

[9] Evans KW, Yuca E, Akcakanat A, Scott SM, Arango NP, Zheng X, Chen K, Tapia C, Tarco E, Eterovic AK (2017). A population of heterogeneous breast cancer patient-derived xenografts demonstrate broad activity of PARP inhibitor in BRCA1/2 wild-type tumors. Clin Cancer Res.

[10] Benjamini Y, Hochberg Y (1995). Controlling the false discovery rate: a practical and powerful approach to multiple testing. JR Statist Soc B.

[11] Iglewicz B, Hoaglin DC (1993). How to Detect and Handle Outliers.

[12] Meric-Bernstam F, Brusco L, Shaw K, Horombe C, Kopetz S, Davies MA, Routbort M, Piha-Paul SA, Janku F, Ueno N (2015). Feasibility of large-scale genomic testing to facilitate enrollment onto genomically matched clinical trials. J Clin Oncol.

[13] Chen K, Meric-Bernstam F, Zhao H, Zhang Q, Ezzeddine N, Tang LY, Qi Y, Mao Y, Chen T, Chong Z (2015). Clinical actionability enhanced through deep targeted sequencing of solid tumors. Clin Chem.

[14] Sangai T, Akcakanat A, Chen H, Tarco E, Wu Y, Do KA, Miller TW, Arteaga CL, Mills GB, Gonzalez-Angulo AM (2012). Biomarkers of response to Akt inhibitor MK-2206 in breast cancer. Clin Cancer Res.

[15] Kalinsky K., Sparano J. A., Zhong X., Andreopoulou E., Taback B., Wiechmann L., Feldman S. M., Ananthakrishnan P., Ahmad A., Cremers S., Sireci A. N., Cross J. R., Marks D. K., Mundi P., Connolly E., Crew K. D., Maurer M. A., Hibshoosh H., Lee S., Hershman D. L. (2018). Pre-surgical trial of the AKT inhibitor MK-2206 in patients with operable invasive breast cancer: a New York Cancer Consortium trial. Clinical and Translational Oncology.

[16] Jonasch E, Hasanov E, Corn PG, Moss T, Shaw KR, Stovall S, Marcott V, Gan B, Bird S, Wang X (2017). A randomized phase 2 study of MK-2206 versus everolimus in refractory renal cell carcinoma. Ann Oncol.

[17] Oki Y, Fanale M, Romaguera J, Fayad L, Fowler N, Copeland A, Samaniego F, Kwak LW, Neelapu S, Wang M (2015). Phase II study of an AKT inhibitor MK2206 in patients with relapsed or refractory lymphoma. Br J Haematol.

[18] Hudis C, Swanton C, Janjigian YY, Lee R, Sutherland S, Lehman R, Chandarlapaty S, Hamilton N, Gajria D, Knowles J (2013). A phase 1 study evaluating the combination of an allosteric AKT inhibitor (MK-2206) and trastuzumab in patients with HER2-positive solid tumors. Breast Cancer Res.

[19] Gupta S, Argiles G, Munster PN, Hollebecque A, Dajani O, Cheng JD, Wang R, Swift A, Tosolini A, Piha-Paul SA (2015). A phase I trial of combined ridaforolimus and MK-2206 in patients with advanced malignancies. Clin Cancer Res.

[20] Chung V, McDonough S, Philip PA, Cardin D, Wang-Gillam A, Hui L, Tejani MA, Seery TE, Dy IA, Al Baghdadi T (2017). Effect of selumetinib and MK-2206 vs oxaliplatin and fluorouracil in patients with metastatic pancreatic cancer after prior therapy: SWOG S1115 study randomized clinical trial. JAMA Oncol.

[21] Ma CX, Suman V, Goetz MP, Northfelt D, Burkard ME, Ademuyiwa F, Naughton M, Margenthaler J, Aft R, Gray R (2017). A phase II trial of neoadjuvant MK-2206, an AKT inhibitor, with anastrozole in clinical stage II or III PIK3CA-mutant ER-positive and HER2-negative breast cancer. Clin Cancer Res.

[22] Triptahy D, Chien AJ, Hylton N, Buxton MB, Ewing CA, Wallace AM, Forero A, Kaplan HG, Nanda R, Albain KS (2015). Adaptively randomized trial of neoadjuvant chemotherapy with or without the Akt inhibitor MK-2206: Graduation results from the I-SPY 2 Trial. J Clin Oncol.

[23] Hyman DM, Smyth LM, Donoghue MTA, Westin SN, Bedard PL, Dean EJ, Bando H, El-Khoueiry AB, Perez-Fidalgo JA, Mita A (2017). AKT inhibition in solid tumors with AKT1 mutations. J Clin Oncol.

[24] Schmid P, Abraham J, Chan S, Wheatley D, Brunt M, Nemsadze G, Baird R, Park YH, Hall P, Perren T (2018). AZD5363 plus paclitaxel versus placebo plus paclitaxel as first-line therapy for metastatic triple-negative breast cancer (PAKT): a randomised, double-blind, placebo-controlled, phase II trial. J Clin Oncol.

[25] Kim SB, Dent R, Im SA, Espie M, Blau S, Tan AR, Isakoff SJ, Oliveira M, Saura C, Wongchenko MJ (2017). Ipatasertib plus paclitaxel versus placebo plus paclitaxel as first-line therapy for metastatic triple-negative breast cancer (LOTUS): a multicentre, randomised, double-blind, placebo-controlled, phase 2 trial. Lancet Oncol.

[26] Yap TA, Yan L, Patnaik A, Tunariu N, Biondo A, Fearen I, Papadopoulos KP, Olmos D, Baird R, Delgado L (2014). Interrogating two schedules of the AKT inhibitor MK-2206 in patients with advanced solid tumors incorporating novel pharmacodynamic and functional imaging biomarkers. Clin Cancer Res.

