# Supplementary figures and images for: Phase II trial of AKT inhibitor MK-2206 in patients with advanced breast cancer who have tumors with PIK3CA or AKT mutations, and/or PTEN loss/PTEN mutation
Source: Breast Cancer Res. 2019 Jul 5;21:78. doi: 10.1186/s13058-019-1154-8 (PMC6612080; doi:10.1186/s13058-019-1154-8)

## Slide 1
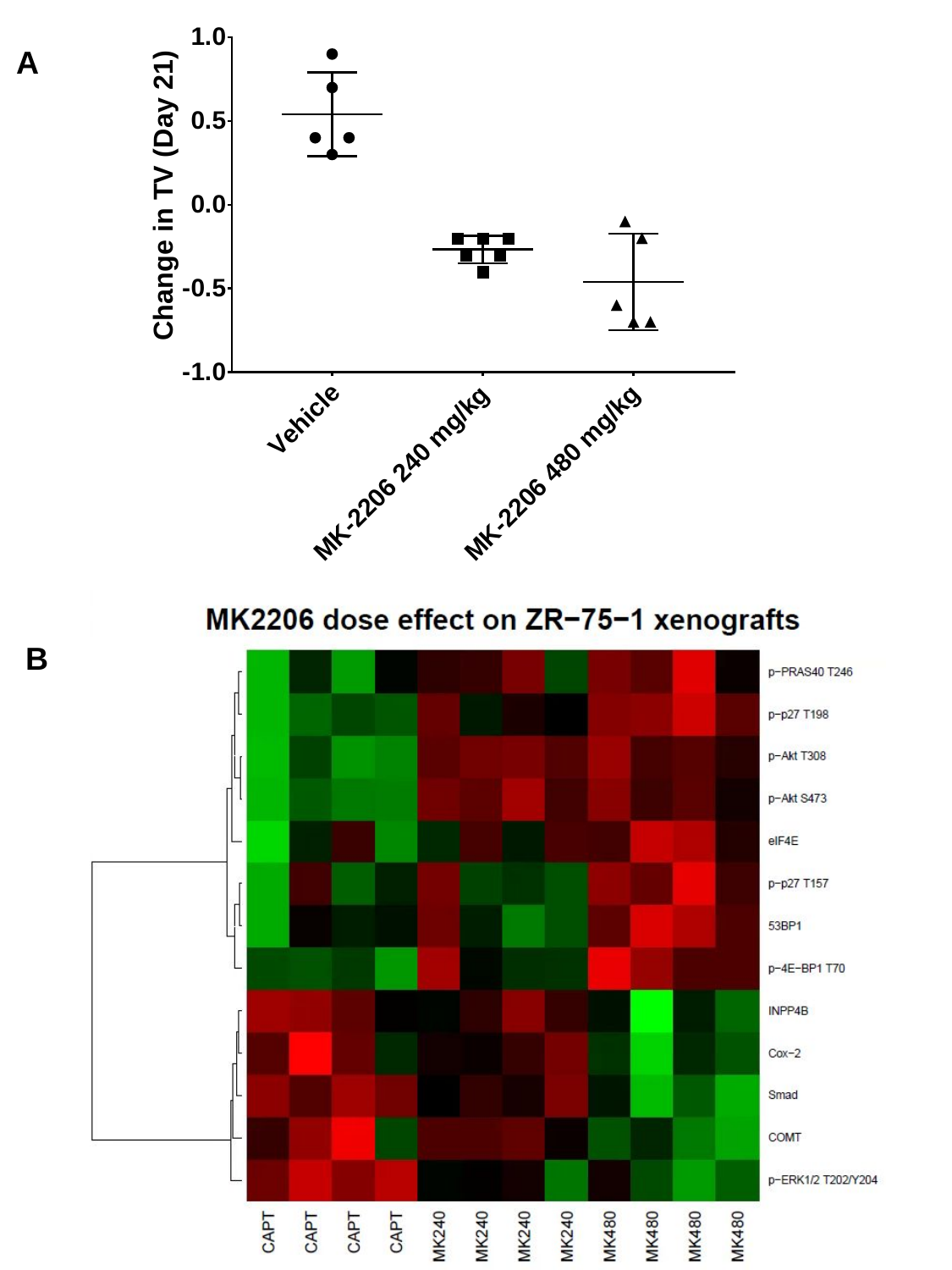

A
B

Supplement: Supplementary file 1 — Figure S1. MK-2206 inhibits tumor growth in ZR75-1 xenografts. A. Scatter plots show the change in tumor volume (TV) calculated for each tumor using the formula (Vf-V0)/V0, where V0 is initial volume (at the beginning of treatment) and Vf is final volume (at the end of treatment). Lines are at mean with SD. Treatment groups (MK-2206 dosed at 240 or 480 mg/kg) were compared with the vehicle (30% Captisol (CYDEX Pharmaceuticals)). B. Heatmap of the 13 significant dose-dependent proteins at the FDR of 0.1 in ZR-75-1 xenografts. (PPTX 130 kb) [file 13058_2019_1154_MOESM1_ESM.pptx]
