# Supplementary material for: Phase II trial of AKT inhibitor MK-2206 in patients with advanced breast cancer who have tumors with PIK3CA or AKT mutations, and/or PTEN loss/PTEN mutation
Source: Breast Cancer Res. 2019 Jul 5;21:78. doi: 10.1186/s13058-019-1154-8 (PMC6612080; doi:10.1186/s13058-019-1154-8)
Supplement: Supplementary file 2 — Table S1. Sequencing data from a 202 gene platform (T200) for two best responders in the study cohort. (DOCX 18 kb) [file 13058_2019_1154_MOESM2_ESM.docx]

**Table S1**: Sequencing data from a 202 gene platform (T200) for two best responders in the study cohort.

| **aaw** | | |  |  |  |  |
| --- | --- | --- | --- | --- | --- | --- |
| **Gene** | **HGNC_AAS** | **Protein Position** | **Codons** | **Amino Acids** | **Exon** | **Allele Freq** |
| EP300 | EP300_S1220F | 1220 | tCc/tTc | S/F | 20/31 | 31.01% |
| EP300 | EP300_M2168I | 2168 | atG/atT | M/I | 31/31 | 34.49% |
| KRAS | KRAS_G12S | 12 | Ggt/Agt | G/S | 2/5 | 33.79% |
| ETV1 | ETV1_Q16E | 16 | Caa/Gaa | Q/E | 1/9 | 26.46% |
| MEN1 | - | 83-84 | - | - | 2/10 | 36.87% |
| GATA3 | - | 437 | - | - | 6/6 | 29.36% |
| PIK3CA | PIK3CA_E542K | 542 | Gaa/Aaa | E/K | 10/21 | 43.83% |
| PTEN | PTEN_D92E | 92 | gaC/gaG | D/E | 5/9 | 5.04% |
| RARA | RARA_T285I | 285 | aCc/aTc | T/I | 7/9 | 33.01% |
| SMARCA4 | SMARCA4_S224L | 224 | tCg/tTg | S/L | 4/38 | 30.08% |
|  |  |  |  |  |  |  |
| **Gene** | **Copy # Alteration** | **Gene** | **Copy # Alteration** |  |  |  |
| CCND1 | H.AMP | ORAOV1 | H.AMP |  |  |  |
|  |  |  |  |  |  |  |
|  |  |  |  |  |  |  |
| **Mutations and Copy Number Alterations for Patient with stable disease** | | |  |  |  |  |
| **Gene** | HGNC_AAS | Protein Position | Codons | **Amino Acids** | **Exon** | **Allele Freq** |
| FANCA | FANCA_D694N | 694 | Gac/Aac | D/N | 23/43 | 21.28% |
| TP53 | TP53_F113C | 113 | tTc/tGc | F/C | 4/12 | 38.18% |
|  |  |  |  |  |  |  |
| **T200 CNA** |  |  |  |  |  |  |
| **Gene** | Copy # Alteration | Gene | Copy # Alteration |  |  |  |
| C19orf12 | H.AMP | CCNE1 | H.AMP |  |  |  |
| FBXO25 | NORM,H.AMP | GPR89A | H.AMP |  |  |  |
| NBPF10 | AMP,H.AMP | NBPF20 | AMP,H.AMP |  |  |  |
| PDZK1 | AMP,H.AMP | TDRP | H.AMP |  |  |  |
| TUSC3 | H.AMP | WNK1 | H.AMP |  |  |  |
